# Vehicle Exhaust Gas Clearance by Low Temperature Plasma-Driven Nano-Titanium Dioxide Film Prepared by Radiofrequency Magnetron Sputtering

**DOI:** 10.1371/journal.pone.0059974

**Published:** 2013-04-03

**Authors:** Shuang Yu, Yongdong Liang, Shujun Sun, Kai Zhang, Jue Zhang, Jing Fang

**Affiliations:** 1 Academy for Advanced Interdisciplinary Studies, Peking University, Beijing, China; 2 College of Engineering, Peking University, Beijing, China; Queen’s University Belfast, United Kingdom

## Abstract

A novel plasma-driven catalysis (PDC) reactor with special structure was proposed to remove vehicle exhaust gas. The PDC reactor which consisted of three quartz tubes and two copper electrodes was a coaxial dielectric barrier discharge (DBD) reactor. The inner and outer electrodes firmly surrounded the outer surface of the corresponding dielectric barrier layer in a spiral way, respectively. Nano-titanium dioxide (TiO_2_) film prepared by radiofrequency (RF) magnetron sputtering was coated on the outer wall of the middle quartz tube, separating the catalyst from the high voltage electrode. The spiral electrodes were designed to avoid overheating of microdischarges inside the PDC reactor. Continuous operation tests indicated that stable performance without deterioration of catalytic activity could last for more than 25 h. To verify the effectiveness of the PDC reactor, a non-thermal plasma(NTP) reactor was employed, which has the same structure as the PDC reactor but without the catalyst. The real vehicle exhaust gas was introduced into the PDC reactor and NTP reactor, respectively. After the treatment, compared with the result from NTP, the concentration of HC in the vehicle exhaust gas treated by PDC reactor reduced far more obviously while that of NO decreased only a little. Moreover, this result was explained through optical emission spectrum. The O emission lines can be observed between 870 nm and 960 nm for wavelength in PDC reactor. Together with previous studies, it could be hypothesized that O derived from catalytically O_3_ destruction by catalyst might make a significant contribution to the much higher HC removal efficiency by PDC reactor. A series of complex chemical reactions caused by the multi-components mixture in real vehicle exhaust reduced NO removal efficiency. A controllable system with a real-time feedback module for the PDC reactor was proposed to further improve the ability of removing real vehicle exhaust gas.

## Introduction

Vehicle exhaust gas contains hydrocarbons, nitrogen oxides, carbon dioxide, carbon monoxide, sulphur dioxide, carbon particles, fine particulate matter and small amounts of aromatic hydrocarbons (benzene) and dioxins. Among these pollutants, hydrocarbons are a major contributor to smog, especially in urban areas. Prolonged exposure to hydrocarbons can cause asthma, liver disease, lung disease and cancer. Carbon monoxide reduces the ability of blood to carry oxygen, and overexposure to carbon monoxide poisoning can be fatal. Nitrogen oxides(NO_x_), which is a mixture of NO, N_2_O, and NO_2_, is generated when nitrogen in the air reacts with oxygen at the high temperature and pressure inside the engine. NO_x_ is a precursor to smog and acid rain.

In the past few decades, non-thermal plasma (NTP) has been widely used to remove volatile organic compounds (VOC) and NO_x_ [1]–[2]. However, the application of NTP has been greatly restricted by its low energy efficiency and poor CO_2_ selectivity. Besides, undesirable byproducts (such as ozone), need to be further treated. Recently, these problems were solved to some extent by a combination of non-thermal plasma with catalyst, so called plasma-driven catalysis. As a promising technology, this technique integrates the advantages of high selectivity from catalysis and fast ignition from plasma, which maintained high energy efficiency and mineralization rate with low by-product formation [3]–[4].

Plasma-driven catalysis (PDC) is a physical and chemical reaction. The reactive species from plasma, such as ions, electrons, excited atomics, molecules and radicals, generate considerable micro-discharge on the surface of the dielectric. These reactive species, especially high energy electrons, contain a large amount of energy, which will activate nearby catalyst and lower the activation energy of the reaction. Internal transition of high-energy particles will generate ultraviolet radiation [5]–[6]. If absorbed energy is greater than the band gap, the electron inside the semiconductor will be excited with a transition from the valence band to the conduction band, which will form electron-hole pairs and induce a series of further redox reactions. Photo-excited holes have a strong ability to obtain electrons, resulting in reacting with the hydroxide ions together with water adsorbed on the catalyst surface and then generating hydroxyl radicals, which leads to a further oxidation of pollutants. Compared with common catalysts, plasma-driven catalysts have many unique advantages, such as high distribution of reactive species, decreased energy consumption, enhanced catalytic activity and selectivity as well as the reduction of the sensitivity to poison [7]–[8].

Compared with non-thermal plasma, the addition of catalysts could significantly enhance the VOC removal efficiency with increased CO_2_ selectivity and carbon balance, while the byproducts, such as O_3_ and organic compounds were dramatically reduced, which was mainly due to increased amount of O formed from O_3_ destruction [9]. Besides, plasma-driven catalysis has also been used in NO_x_ removal. It was reported that (plasma generated) ozone, hydroxyl radicals and atomic oxygen played important roles in the oxidation of NO to NO_2_ [10]_._ Many efforts have been made to purify VOC or NO_x_ gas by using plasma-driven catalysis [11]–[13]. However, those studies focused on only one specific polluted gas or some simulated gases, which were quite different from real vehicle exhaust gas from a launched car. In fact, complex components in the exhaust interacted with each other. For example, according to the molecular dynamics theory, NO removal efficiency depends heavily on the content of HC and O_2_ [14]. Meanwhile, partially oxidized hydrocarbons and peroxy radicals (RO_2_) will in return react with NO and strongly influence NO_2_ formation rates [10]. Thus the application of the technique for examining the vehicle exhaust removal rate has a practical significance.

In previous studies, catalyst material can be introduced into the reactor in several ways, such as coating on the reactor wall or electrodes, as a packed-bed (granulates, coated fibers, pellets) or as a layer of catalyst material (powder, pellet, granulates, coated fiber) [8]. What researches worried about was the deactivation of catalyst [15], [16]. The catalyst contacted with the high electrode directly in all the studies above. What’s more, most catalysts were prepared with liquid phase method, which contained too many complex chemical steps, even toxic or organic gas evaporating into the air [16], [17], [18], [19].

In this study, a novel and special structure plasma-driven catalysis device was proposed. The PDC reactor was a coaxial DBD reactor with three dielectric barrier layers and two copper electrodes. The middle dielectric barrier was designed to separate the catalyst from the high voltage electrode. The catalyst TiO_2_, prepared by RF magnetron sputtering, was coated on the outer surface of the middle quartz tube. The electrodes surrounded the outer surfaces of corresponding dielectric barrier in a spiral way to prevent the damage to the catalyst for too much heat from microdischarges in PDC reactor. Then the PDC reactor was employed to treat the real vehicle exhaust. In order to explain the result, a simple and intuitive method–optical emission spectroscopy was conducted, which was different from chemical kinetics analysis in previous studies. This study also analyzed the practical problems for the application of PDC technique in real vehicle exhaust and proposed some solutions.

## Materials and Methods

### The PDC Reactor

**Figure 1:**
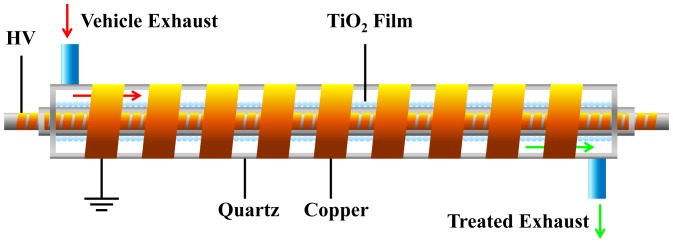
A schematic diagram of plasma-driven catalysis reactor. The PDC reactor was a coaxial DBD reactor with three quartz tubes as dielectric barrier layers and two copper electrodes. The outer and inner electrodes attached to the surfaces of the corresponding quartz tubes in a spiral rotation, respectively. The length of the whole reactor was 180 mm. The outside diameters of the quartz tubes were 18 mm, 10 mm and 6 mm, respectively.

**Figure 2:**
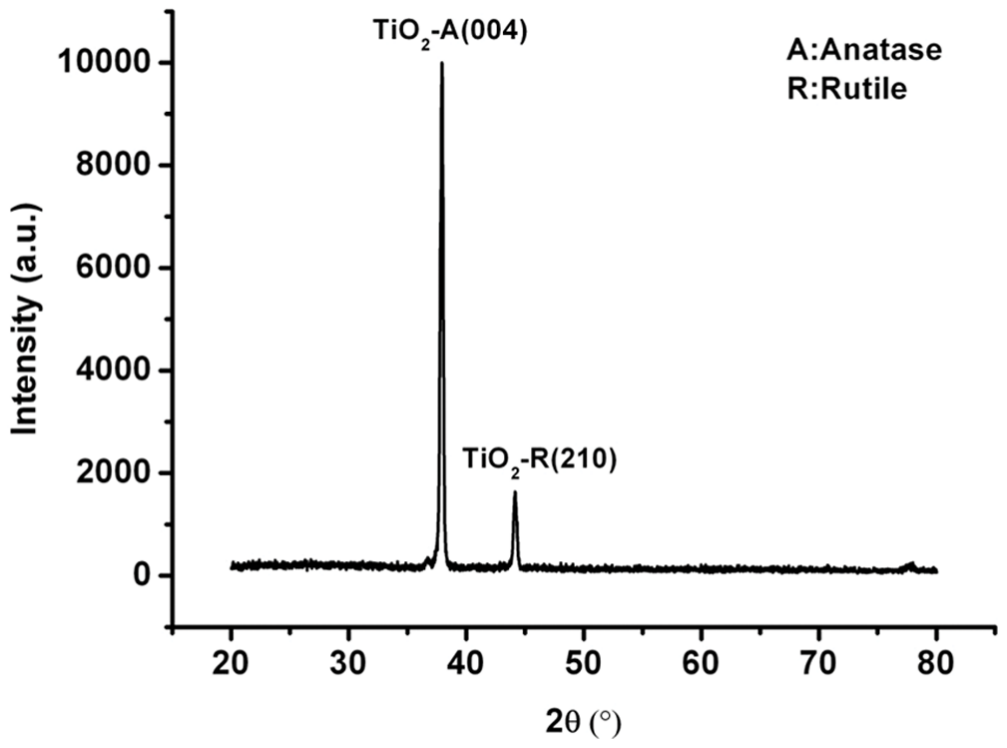
XRD pattern of prepared TiO_2_ film. The titanium dioxide has an approximate composition of 85% anatase and 15% rutile forms of TiO_2_.

**Figure 3:**
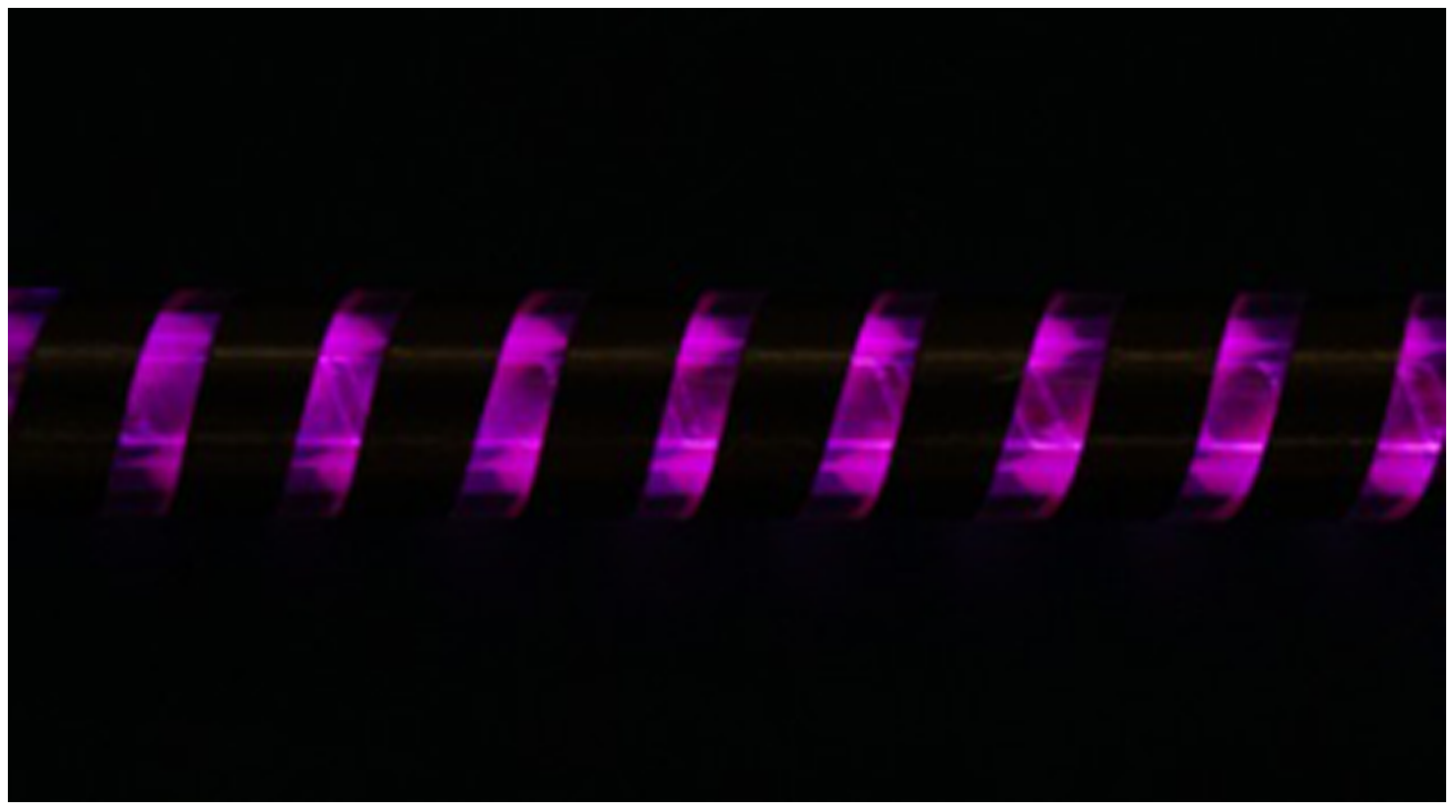
Discharge of plasma-driven catalysis reactor. The excited voltage and power were about 7.78 kV and 2.75W, respectively.

**Figure 4:**
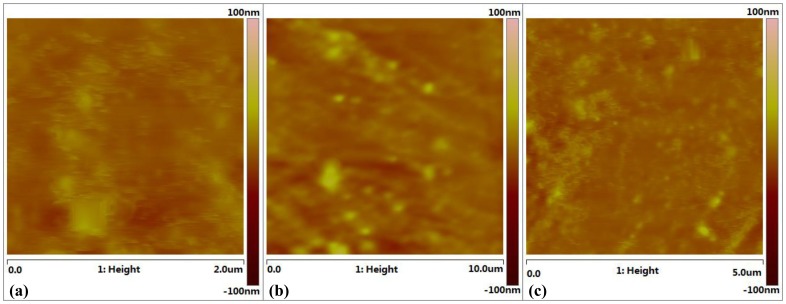
AFM photograph of prepared TiO_2_ film. AFM detections on three points on the middle quartz tube with different distances of (a) 20 mm, (b) 50 mm and (c) 90 mm from the same side.

As shown in Fig. 1, the proposed PDC reactor was a coaxial DBD reactor with three quartz tubes as dielectric barrier layers and two copper electrodes. The inner electrode attached to the inner quartz tube with a spiral rotation, of which the helix width and the pitch were 6 mm and 2 mm, respectively, while the outer electrode attached to the outer quartz tube in the same way as the helix width of 9 mm and the pitch of 4 mm. The nano-titanium dioxide film prepared by radio frequency magnetron sputtering (JGP450 High vacuum magnetron sputtering, China) was coated on the outer surface of middle quartz tube. During the process of coating TiO_2_ film, the Ti target was set at the bottom of the vacuum chamber to avoid a large amount of impurities groups into TiO_2_ film, and the inner quartz rotated by a constant angular velocity of 0.15 r/s, guaranteeing a well-distributed film on the same circle of the reactor. The proportion of oxygen and argon was about 1∶1 with the power of 150W at 1 Pa pressure. The XRD detection given in Fig. 2 indicates that the titanium dioxide has an approximate composition of 85% anatase and 15% rutile forms of TiO_2_. The discharge of the whole PDC reactor was shown in Fig. 3, with the excited voltage of 7.78 kV and the power of 2.75W. In order to know the surface features of the TiO_2_, AFM detection was conducted on three points on the middle quartz tube with different distances of 20 mm, 50 mm, 90 mm from the same side, respectively. It was worth mentioning that the tube rotated at a certain speed of 0.15 r/s, which would help to form the uniform film in the same circle. Fig. 4 shows the AFM photograph of the uniform surface of the TiO_2_. The length of the whole PDC reactor was 180 mm, and the outside diameter of the outer quartz tube was 18 mm, which constituted a compact reactor.

### Clearance System

In this study, the experiment included exhaust gas source collection, treatment and components detection. The real exhaust gas was acquired from a jeep (Charokee-type Beijing Jeep 2500, made in China in 1999), in which the engine with inline four-cylinder was fed by #93 gasonline, in the condition of temperature/humidity 90°C/60% (monitored by MINGLE Hygrometer TH101B, China). The contents of components in the vehicle exhaust before and after treatment with the PDC reactor were detected by an exhaust gas analyzer (CV-5Q, Tianjin Shengwei Inc. Tianjin, China), which serves officially as a standard exhaust analysis in Beijing, China.

### Clearance Process

**Figure 5:**
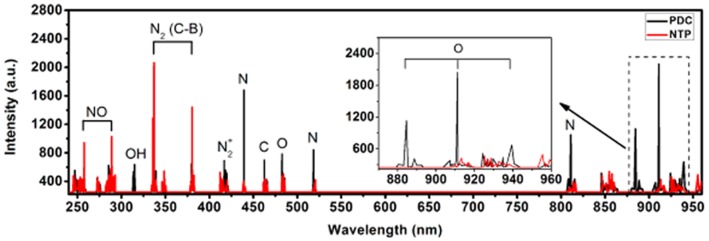
Spectrum of the discharge by PDC and NTP reactor. The appearance of O spectrum can be observed between 870 nm and 960 nm for wavelength in PDC reactor.

At the beginning of each experiment, the jeep was started to let the vehicle exhaust gas steadily emit while the exhaust gas analyzer was turned on for detection. Then the exhaust was induced into the PDC reactor, which was excited for low temperature plasma later at the voltage of 8–10 kV. As shown by the detected spectrum in Fig. 5, many significant peaks can be observed in spectrum scope of working plasma inside the PDC reactor ranged from 250 nm to 520 nm, which satisfies the required wavelengths of TiO_2_ catalysis.

We then conducted experimental comparisons among three different groups, including a control group without any plasma treatment, a PDC group with TiO_2_, and a NTP group which had an identical structure to the PDC reactor but without the catalyst TiO_2_. Five tests were conducted for each group. In each test, after engine reaching steady state, the concentrations of different components of the vehicle exhaust were recorded sequentially at an interval of 20 s within 3 minutes for three groups. Then plasma was excited in both PDC and NTP reactors which the vehicle exhaust gas was lead into. The concentrations of different components were recorded at an interval of 20 s within another 9 minutes for these two groups.

### Electrical Measurement

The PDC reactors were ignited by an AC high voltage power supply equipped with a transformer, which controlled the input power of the plasma generator (CTP-2000K). AC high frequency high voltage exported from the generator was applied to the PDC reactor, providing excitation power for the reactor. The excitation power was measured through the output voltage and current detection in the generator. Applied high voltage (V) was measured with a 1000∶1 high voltage probe (TEKtronix, P6015A). V–Q Lissajous method was used to determine the discharge power in the PDC reactor. The charge Q was determined by measuring the voltage across the capacitor of 0.47 uF connected in series to the ground line of the PDC reactor. The voltage across this capacitor is proportional to the charge. The signals of applied voltage and charge were recorded with a digitizing oscilloscope (Tektronix, MSO2024) by averaging 62.5 k scans. The discharge power (P_dis_) was evaluated from the area of V–Q parallelogram by multiplying the frequency. Specific input energy (SIE), which is defined as the energy input per unit gas-flow rate, can be obtained as follows:
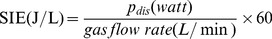
(1)


In this study, both the minimum excited state and steady state of working PDC reactor were measured according to the method above.

### Optical Emission Spectroscopy

Optical emission spectroscopy (OES) is one of the most widely used diagnostic methods for low-temperature plasmas [20]. Optical emission spectrometry involves applying electrical energy in the form of spark generated between an electrode and a metal sample, whereby the vaporized atoms are brought to a high energy state within a so-called “discharge plasma”. These excited atoms and ions in the discharge plasma create a unique emission spectrum specific to each element. Thus, a single element generates numerous characteristic emission spectral lines. Therefore, the light generated by the discharge can be said to be a collection of the spectral lines generated by the elements in the sample. This light is split by a diffraction grating to extract the emission spectrum for the target elements. The intensity of each emission spectrum depends on the concentration of the element in the sample. Detectors (photomultiplier tubes) measure the presence or absence of the spectrum extracted for each element and the intensity of the spectrum to perform qualitative and quantitative analysis of the elements [21], [22]. The vehicle exhaust gas was induced into the PDC and NTP reactors, respectively. And the plasmas in the two reactors were excited with the same voltage and power. The probe was put at the same point of each reactor.Then the spectrums were obtained.

## Results

### Vehicle Exhaust Clearance

**Figure 6:**
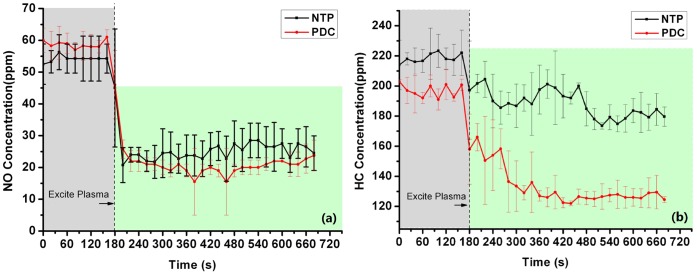
Real-time removal results of (a) NO removal and (b) HC removal. The removal efficiency for HC in the PDC reactor wasη_NO_ = 64.5%±1.8%, and the removal efficiency for NO in the PDC reactor wasη_HC_ = 32.1%±1.3%



Figure 6(a) and (b) show that both of the two reactors have a positive effect on the reduction of HC and NO. Moreover, the removal efficiency of PDC reactor was significantly higher than that of NTP reactor. The vehicle exhaust removal efficiency η was estimated as follow:

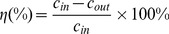
(2)where c_in_, c_out_ are inlet and outlet concentration of the certain component, respectively.

According to Eq.1, the removal efficiency in the PDC reactor for HC and NO are as follows:

η_NO_ = 64.5%±1.8%, and η_HC_ = 32.1%±1.3%.

### Electrical Measurement

**Figure 7:**
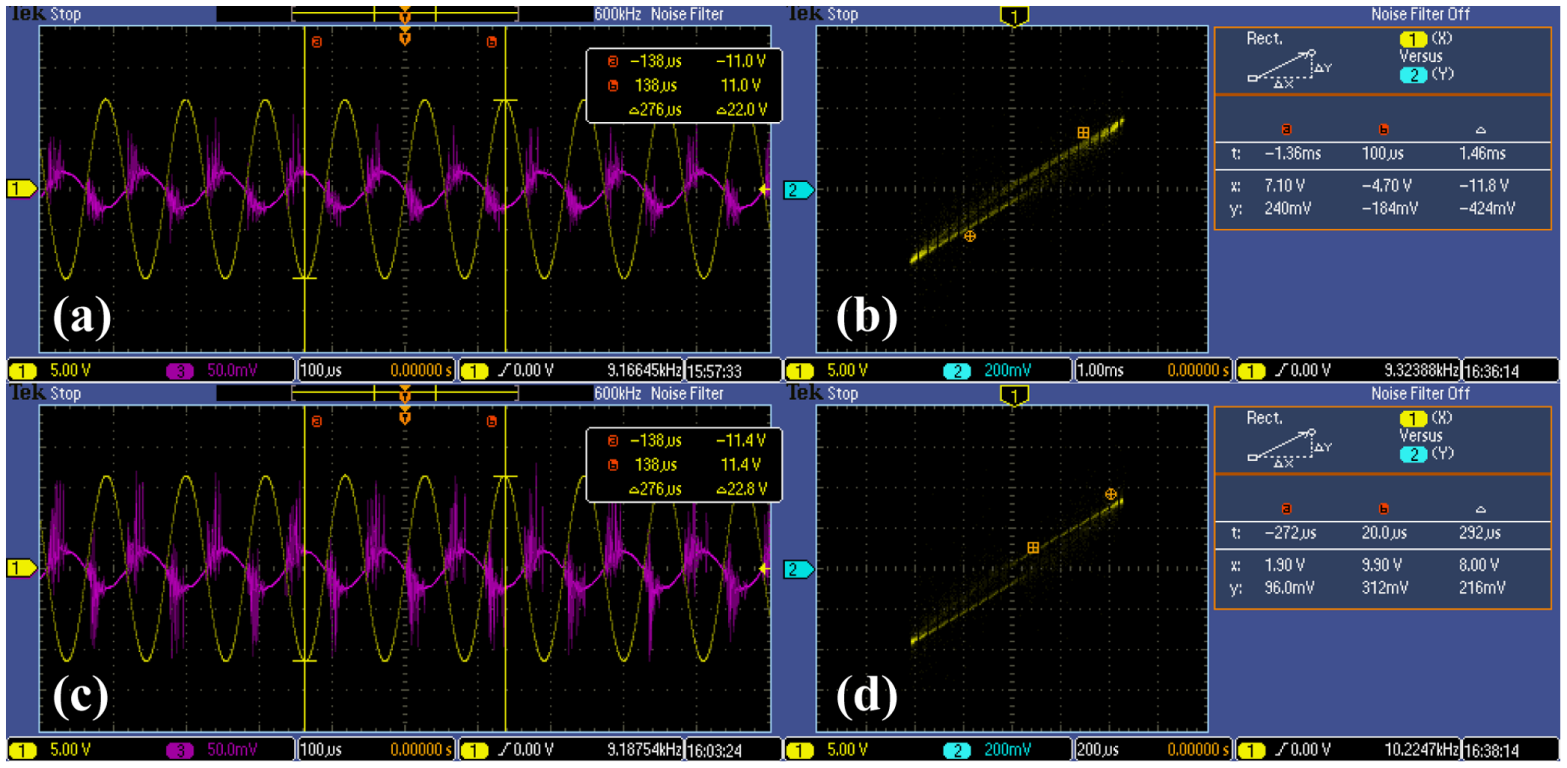
Electrical measurement of the PDC reactor. (a) The voltage and current of the PDC reactor at the minimum excited state. (b) Lissajous figure of the PDC reactor at the minimum excited state. (c) The voltage and current of the PDC reactor at the stable working state. (d) Lissajous figure of the PDC reactor at the stable working state.

Experimental data indicated that our vehicle exhaust flow rate was about 590 sccm. The voltage and current of the PDC reactor were measured through capacitor sampling and resistance sampling, respectively. The V-Q Lissajous method was used to determine the discharge power. Both of the minimum excited state and steady state of PDC reactor were measured. All the electrical measurement results were shown in Fig. 7. At the minimum excited state, the effective voltage and current were 7.78 kV and 0.35 mA. Specific input energy was about 279.66 J/L. At the steady state, the effective voltage and current were 8.06 kV and 0.35 mA. Specific input energy was about 289.83 J/L.

### Optical Emission Spectrum

The optical emission spectrum result was shown in Fig. 5. The OH, NO and N emission lines are visible. The appearance of O emission lines can be observed between 870 nm and 960 nm for wavelength in PDC reactor [23], [24], [25], [26]. Considering the facts that the removal efficiency of HC treated by PDC was much higher than that of NTP, while the removal efficiency of NO by PDC was only a little higher than that of NTP, we hypothesized that O might have made a significant contribution to the much higher HC and NO removal efficiencies.

## Discussion

First of all, in this study, the proposed PDC reactor was designed with three quartz tubes as dielectric layers. The outer and middle quartz tubes were both dielectric barrier layers. The middle one could separate the catalyst from the high voltage electrode, which could prevent high voltage electrode from being oxidized by oxygen during the reactions. On the other hand, the middle quartz tube could increase the area for depositing more TiO_2_ film compared with a bare electrode. Furthermore, the addition of middle quartz tube could provide more chances to generate microdischarges [27], which would increase catalyst surface temperature [28], enhance the dispersion of active catalytic components [29], [30] and influence the stability with catalytic activity of the exposed catalyst material [31]. All above would promote catalytic VOC removal efficiency. However, hot spots can be formed in PDC reactors as a result of localizing heating by intense microdischarges, which might lead to the damage to the high voltage electrode and catalyst [32]. In order to avoid too many hot spots, each electrode surrounded the outer surfaces of corresponding dielectric barrier in a spiral way. The method of preparing TiO_2_ film was RF magnetron sputtering without any toxic or organic gas evaporating into the air. Continuous operation tests indicated that stable performance without deterioration of catalytic activity could last for more than 25 h.

Secondly, the removal efficiency result was further explained through the optical emission spectrum approach, a simple and intuitive method different from chemical kinetics analysis in previous studies.The optical emission spectrum result showed that the appearance of O emission lines can be observed between 870 nm and 960 nm for wavelength in PDC reactor. It is our suggestion that the enhanced performance of hydrocarbon destruction was mainly due to a great amounts of atomic oxygen (O) formed, primarily from catalytically O_3_ decomposition. Compared with NTP reactor, TiO_2_ film acting as a semiconductor oxide catalyst provided a large number of free electron-hole pairs and consequently promoted the oxidation-reduction reaction.

The emission of O_3_ from the NTP reactor was harmful to both human health and global environment. The addition of catalyst could significantly enhance the HC destruction with an increased O formation while the byproducts O_3_ from the plasma were dramatically reduced [9]. Basically, O_3_ formation in the NTP reactor proceeded via a two-step process [33]: formation of atomic oxygen and recombination of atomic oxygen with oxygen molecule (Eqs. (3)-(5)):
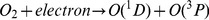
(3)

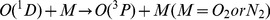
(4)

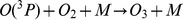
(5)where O(^1^D) and O(^3^P) represent the excited and ground state oxygen atom, respectively.

It has been also reported that O_3_ can be decomposed by catalysts into molecular oxygen via atomic oxygen and peroxides (Eqs. (6)-(8)) [34], where * denotes an active site on the catalyst surface:
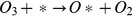
(6)

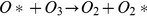
(7)

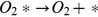
(8)


In general, atomic oxygen, which is highly active and involved in HC oxidation, is also imposed positive effect on NO destruction [9].

Thirdly, in the presence of catalyst TiO_2_ film, when there is a faster rate of oxidation of hydrocarbons (Fig. 6b) there is not a significant increasing reduction of NO (Fig. 6a) correspondingly. Since the gas employed in this study was real vehicle exhaust gas containing many different kinds of compositions, there would be some reactions that did not occur in only one specific gas or some simulated gases. Actually, those complex components in the vehicle exhaust interacted with each other during the PDC process, which would remarkably influence the removal efficiency of NO and HC. It is worth mentioning that there exists a dynamic equilibrium between NO and HC, that HC decomposition will lead to the formation of NO [14]. Besides, it is known that both •OH and O_3_ play an important role in NO removal. However, since O_3_ is an •OH scavenger, partial O_3_ and •OH will react with each other as follow [35]:
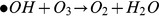
(9)


Thus both contents of O_3_ and •OH will decrease with an increased O_2_ content. It has been reported in a previous literature that high O level will definitely lead to a conversion back to NO, and decrease the the removal efficiency of NO [36]. Although several studies have indicated that plasma-driven catalysis technique was quite effective in removing NO or hydrocarbon [37], [38], [39], [40], [41], according to our results and analysis above, the clearance rate of NO would be reduced if mixed with HC as well as other gas components.

**Figure 8:**
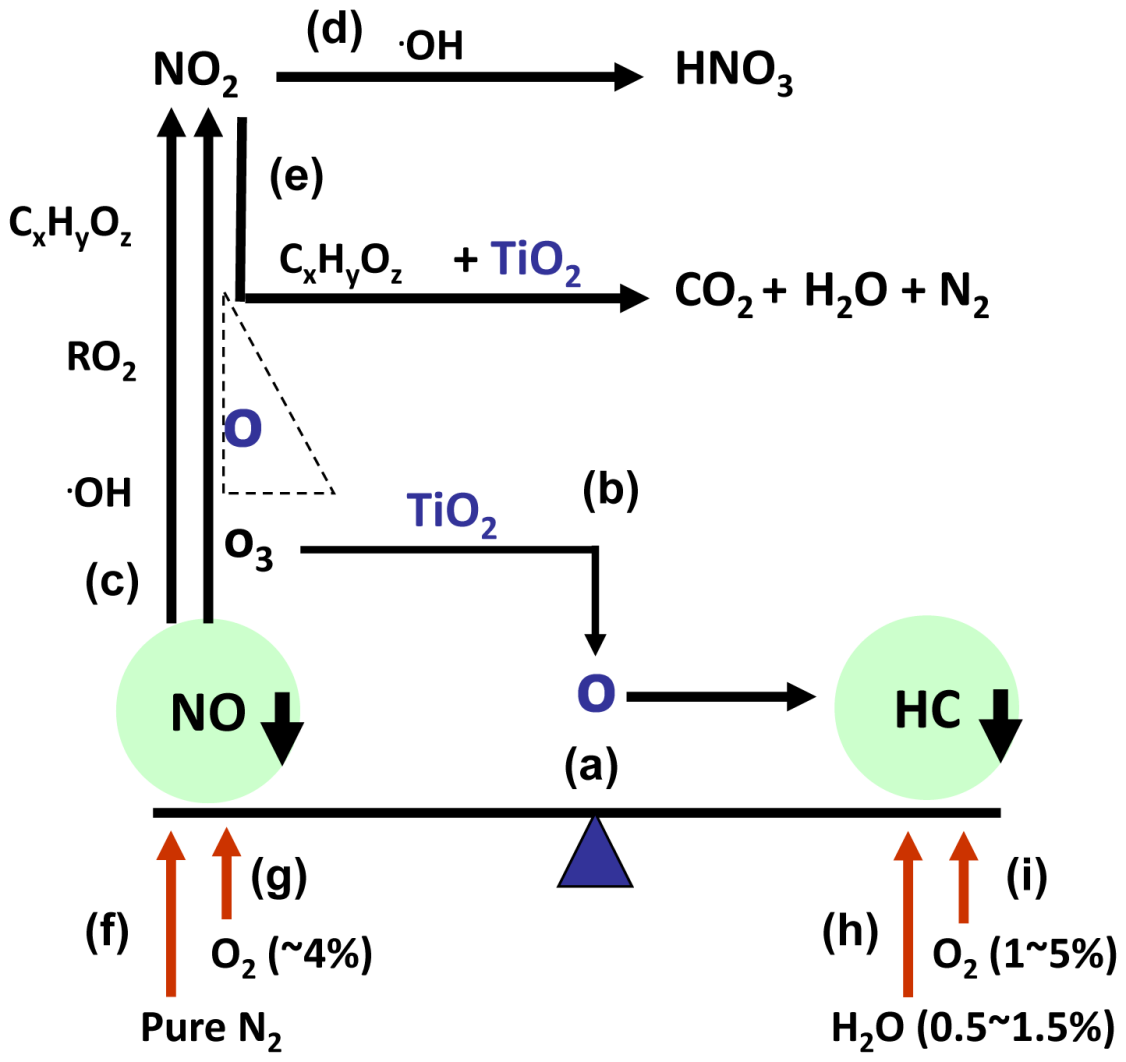
Chemical reactions gas compositions in the vehicle exhaust gas. (a) HC decomposition will lead to the formation of NO [14]. (b) The addition of catalysts could generate more single atomic oxygen from O_3_ destruction, which contributed to the HC decomposition [9]. (c) single atomic oxygen, ozone, OH, oxidized hydrocarbons and peroxyl radicals played an important role in the oxidation of NO to NO_2_ [10], and high O level will definitely lead to a conversion back to NO [36]. (d) •OH radicals can convert the formed NO_2_ into HNO_3_ [10] with the existence of H_2_O [9]. (e) NO_2_ reacted with the catalyst TiO_2_ film, while partially oxidized hydrocarbons is consumed during selective catalytic reduction, producing CO_2_, N_2_, and H_2_O. (f) Nitrogen in air can help keep the high removal efficiency of NO [45]. (g) NOx decomposition by plasma was known to be possible only if the oxygen content was less than about 4% [45], [46]. (h) The highest carbon balance and CO_2_ selectivity for HC destruction were obtained with water vapor content between 0.5 and 1.5% [24]. (i) The optimal oxygen ranges between 1% and 5% for VOC with NTP [44].



The complex chemical reactions among gas compositions in the vehicle exhaust gas are briefly illustrated in Fig. 8. For example, higher NO removal efficiency is under the condition of lower content of HC or decreased O_2_ content [14], meanwhile, NO can be removed by the formation of NO_2_ through the reaction with partially oxidized hydrocarbons and peroxyl radicals (RO_2_) [10]. Thus, the removal efficiency of HC could not be as high as that of NO in our experiment. Besides, many fundamental components from working plasma, including ozone, hydroxyl radicals and atomic oxygen also play an important role in the oxidation of NO to NO_2_ [10]. When hydrocarbon was treated by the plasma discharge, partially oxidized hydrocarbons (C_x_H_y_O_z_) and peroxy radicals (RO_2_) reacting with NO will be generated and strongly influenced NO_2_ formation rate. Meanwhile, NO_2_ reacted with the catalyst TiO_2_ film, while partially oxidized hydrocarbons were consumed during selective catalytic reduction, producing CO_2_, N_2_, and H_2_O, which are environment-friendly products [42]. Finally, •OH radicals can convert the formed NO_2_ into HNO_3_ [10] with the existence of H_2_O [9]:

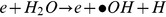
(10)

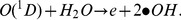
(11)


Based on the complex chemical reactions mentioned above, the removal efficiency of NO and HC should have been much higher if the PDC reactor was used to remove only NO or HC at the same amount of electricity consumption.

Besides, there were some other factors affecting the removal efficiency of NO and HC by using the proposed PDC reactor. On one hand, water vapor existing in the vehicle exhaust would reduce the vehicle exhaust removal efficiency. Although the drying modules had been assembled in our experiments, water vapor could not be removed completely and it would generate a considerable number of •OH, leading to enhancement of NO conversion but decrease of HC removal efficiency with an increased incompletely oxidizing byproducts [10]. Meanwhile, water vapor can make catalyst deactivate through poisoning its active sites, annihilating high energetic electrons and depress the HC destruction through competing to be absorbed by the catalyst [43]. It has been reported that the highest carbon balance and CO_2_ selectivity were obtained with water vapor content between 0.5 and 1.5% [17]. In further study, water vapor could be well controlled to further improve the exhaust removal efficiency.

**Figure 9:**
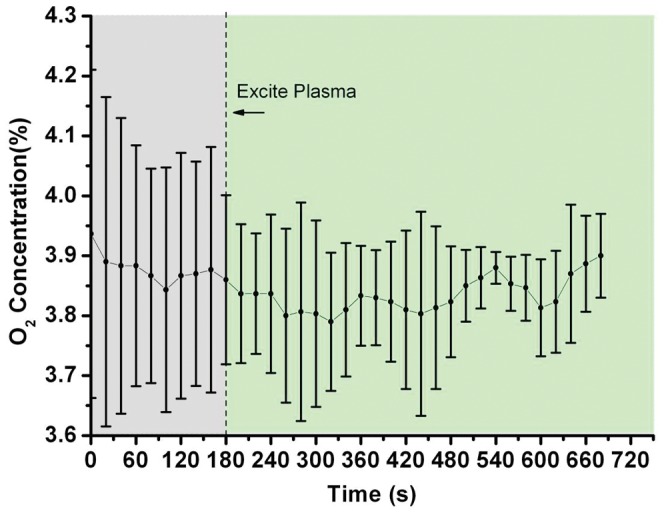
The O_2_ content in the PDC reactor during the whole process. The oxygen content in the exhaust ranged roughly from 3.5% to 4.2% all the time.

On the other hand, similar to the presence of water vapor, the oxygen content in the vehicle exhaust that affects significantly the discharge performance plays a key role in the occurring chemical reactions. It has been mentioned that the optimal oxygen ranges between 1% and 5% for VOC with NTP [44], while NO_x_ decomposition by plasma was known to be possible only if the oxygen content was less than about 4% [45], [46]. In this study, the oxygen content in the exhaust ranged roughly from 3.5% to 4.2% all the time as shown in Fig. 9, which could benefit the PDC reactor for HC removal. However, the oxygen content above could have also benefited the PDC reactor for NO removal efficiency, but it was unreal for the exhaust gas containing VOC [14]. The effect of oxygen content on the NO and HC removal efficiency in this study was obviously reflected in the removal result as shown in Fig. 6.

In order to enhance the ability of the application of PDC reactor in vehicle exhaust gas, a reliable automatic control system will be expected. Based on the measured proportion of HC and NO in real-time, vehicle exhaust can be further removed effectively by the feedback control system that adaptively induces the air outside into PDC reactor. Particularly, the oxygen in air can be used to modulate the appropriate proportions among HC, NO and O_2_ while the nitrogen in air can help keep the high removal efficiency of NO [45]. In this way, the real vehicle exhaust gas from cars will be well controlled with high efficiency in real-time.

## References

[1] Mista W, Kacprzyk R (2008) Decomposition of toluene using non-thermal plasma reactor at room temperature. Catal. Today 137: 345–349.

[2] Song CL, Bin F, Tao ZM, Li FC, Huang QF (2009) Simultaneous removals of NO_x_, HC and PM from diesel exhaust emissions by dielectric barrier discharges. J. Hazard. Mater. 166: 523–349. 10.1016/j.jhazmat.2008.11.068 19128874

[3] Chen HL, Lee HM, Chen SH, Chang MB, Yu SJ, et al (2009) Removal of Volatile Organic Compounds by Single-Stage and Two-Stage Plasma Catalysis Systems: A Review of the Performance Enhancement Mechanisms, Current Status, and Suitable Applications. Environ. Sci. Technol (43) 2216–2227. 10.1021/es802679b 19452866

[4] Fan X, Zhu T, Wang M, Li X (2009) Removal of low-concentration BTX in air using a combined plasma catalysis system. Chemosphere 75: 1301–1306. 19375149 10.1016/j.chemosphere.2009.03.029

[5] Francke KP, Miessner H, Rudolph R (2000) Plasmacatalytic processes for environmental problems. Catal. Today 59: 411–416.

[6] Futamura S, Zhang A, Einaga H, Kabashima H (2002) Involvement of catalyst materials in nonthermal plasma chemical processing of hazardous air pollutants. Cataly. Today 72: 259–265.

[7] Chen HL, Lee HM, Chen SH, Chao Y, Chang MB (2008) Review of plasma catalysis on hydrocarbon reforming for hydrogen production–Interaction, integration, and prospects. Appl. Phys. B: Environmental 85: 1–9.

[8] Durme JV, Dewulf J, Leys C, Langenhove HV (2008) Combining non-thermal plasma with heterogeneous catalysis in waste gas treatment: A review. Appl. Phys. B: Environmental 78: 324–333.

[9] Huang HB, Ye DQ, Dennis YCL (2011) Abatement of Toluene in the Plasma-Driven Catalysis: Mechanism and Reaction Kinetics. IEEE Trans. Plasma Sci. 39: 877–882.

[10] Maciuca A, Dupeyrat CB, Tatibouët JM (2008) Synergetic effect by coupling photocatalysis with plasma for low VOCs concentration removal from air. Appl. Catal. B: Environ. 125: 432–438.

[11] Rappe KG, Hoard W, Aardahl CL, Park PW, Peden CHF, et al (2004) Combination of low and high temperature catalytic materials to obtain broad temperature coverage for plasma-facilitated NO_x_ reduction. Catal. Today 89: 143–150.

[12] Oh SM, Kim HH, Einaga H, Ogata A, Futamura S, et al (2006) Zeolite-combined plasma reactor for decomposition of toluene. Thin Solid Films 506: 418–422.

[13] Holzer F, Roland U, Kopinke FD (2002) Combination of non-thermal plasma and heterogeneous catalysis for oxidation of volatile organic compounds: Part 1. Accessibility of the intra-particle volume. Appl. Catal. B: Environ. 32: 163–181.

[14] Kima HH, Ogat A (2011) Nonthermal plasma activates catalyst: from current understanding and future prospects. Phys. J. Appl. Phys. 55, 13806.

[15] Harling AM, Kim HH, Futamura S, Whitehead J C (2007) Temperature Dependence of Plasma-Catalysis Using a Nonthermal, Atmospheric Pressure Packed Bed; the Destruction of Benzene and Toluene. J. Phys. Chem. C 2007, 111, 5090–5095.

[16] Kim HH, Oh SM, Ogata A, Futamura S (2004) Decomposition of benzene using Ag/TiO2 packed plasma-driven catalyst reactor: influence of electrode configuration and Ag-loading amount. Catalysis Letters Vol. 96, Nos. 3–4, July.

[17] Huang HB, Ye DQ, Dennis YCL (2011) Plasma-Driven Catalysis Process for Toluene Abatement: Effect of Water Vapor. IEEE Trans. Plasma Sci. 39: 576–580.

[18] Ding HX, Zhu AM, Lu FG, Xu Y, Zhang J, et al (2006) Low-temperature plasma-catalytic oxidation of formaldehyde in atmospheric pressure gas streams. J. Phys. D: Appl. Phys. 39: 3603–3608.

[19] Harling AM, Demidyuk V, Fischer SJ, Whitehead JC (2008) Plasma-catalysis destruction of aromatics for environmental clean-up: Effect of temperature and configuration. Applied Catalysis B: Environmental 82: 180–189.

[20] Zhu X, Pu Y (2010) Optical emission spectroscopy in low-temperature plasmas containing argon and nitrogen: determination of the electron temperature and density by the line-ratio method. J. Phys. D: Appl. Phys. 43: 403001.

[21] Fantz U (2006) Basics of plasma spectroscopy. Plasma Sources Sci. Technol. 15: S137–S147.

[22] Staack D, Farouk B, Gutsol AF, Fridman AA (2006) Spectroscopic studies and rotational and vibrational temperature measurements of atmospheric pressure normal glow plasma discharges in air. Plasma Sources Sci. Technol. 15 (2006) 818–827.

[23] Walsh JL, Kong MG (2008) Contrasting characteristics of linear-field and cross-field atmospheric plasma jets. Appl. Phys. Lett. 93, 111501.

[24] Lee YH, Yi CH, Chung MJ, Yeom GY (2001) Characteristics of He/O_2_ atmospheric pressure glow discharge and its dry etching properties of organic materials. Surf. Coat. Technol. 146–147: 474–479.

[25] Lofthus A, Krupenie PH (1977) The spectrum of molecular nitrogen. J. Phys. Chem. Ref. Data 6, 113.

[26] Xu L, Nonaka H, Zhou HY, Ogino A, Nagata T, et al (2007) Characteristics of surface-wave plasma with air-simulated N_2_–O_2_ gas mixture for low-temperature sterilization. J. Phys. D: Appl. Phys. 40: 803–808.

[27] Kogelschatz U (2003) Dielectric-barrier Discharges: Their History, Discharge Physics, and Industrial Applications. Plasma Chem. Plasma Process.Vol. 23, No. 1, March.

[28] Lu B, Zhang X, Yu X, Feng T, Yao S (2006) Catalytic oxidation of benzene using DBD corona discharges, Journal of Hazardous Materials. 137: 633–637. 10.1016/j.jhazmat.2006.02.012 16621276

[29] Guo YF, Ye DQ, Chen KF, He JC, Chen WL (2006) Toluene decomposition using a wire-plate dielectric barrier discharge reactor with manganese oxide catalyst in situ. Journal of Molecular Catalysis A: Chemical 245: 93–100.

[30] Zhang YP, Ma PS, Zhu XL, Liu CJ, Shen YT (2004) A novel plasma-treated Pt/NaZSM-5 catalyst for NO reduction by methane, Catalysis Communications. 5: 35–39.

[31] Guo YF, Ye DQ, Chen KF, He JC (2007) Toluene removal by a DBD-type plasma combined with metal oxides catalysts supported by nickel foam, Catalysis.

[32] Kim HH, Ogata A, Futamura S (2006) Effect of different catalysts on the decomposition of VOCS using flow-type plasma-driven catalysis, IEEE Transactions on Plasma Science. 34: 984–995.

[33] H. H Kim, A Ogata, S Futamura (2006) Effect of different catalysts on the decomposition of VOCs using flow-type plasma-driven catalysis, IEEE Trans. Plasma Sci. 34: 984–995.

[34] Futamura S, Einaga H, Kabashima H, Hwan LY (2004) Synergistic effect of silent discharge plasma and catalysts on benzene decomposition. Catal. Today 89: 89–95.

[35] Kim HH, Oh SM, Ogata A, Futamura S (2005) Decomposition of gas-phase benzene using plasma-driven catalyst (PDC) reactor packed with Ag/TiO2 catalyst. Appl. Catal. B Environ (56) 213–220.

[36] Malik MA, Kolb JF, Sun Y, Schoenbach KH (2011) Comparative study of NO removal in surface-plasma and volume-plasma reactors based on pulsed corona discharges. J. Hazard. Mater. 197: 220–228. 10.1016/j.jhazmat.2011.09.079 21982539

[37] X Tu, J.C Whitehead (2012) Plasma-catalytic dry reforming of methane in an atmospheric dielectric barrier discharge: Understanding the synergistic effect at low temperature. Appl. Catal. B. 125: 439–448.

[38] Fan HY, Shi C, Li XS, Zhao DZ, Xu Y, et al (2009) High-efficiency plasma catalytic removal of dilute benzene from air. J. Phys. D: Appl. Phys. 42: 225105.

[39] Ding HX, Zhu AM, Lu FG, Xu Y, Zhang J, et al (2006) Low-temperature plasma-catalytic oxidation of formaldehyde in atmospheric pressure gas streams. J. Phys. D: Appl. Phys. 39: 3603–3608.

[40] Mok YS, Koh DJ, Kim KT, Nam IS (2003) Nonthermal Plasma-Enhanced Catalytic Removal of Nitrogen Oxides over V_2_O_5_/TiO_2_ and Cr_2_O_3_/TiO_2_. Ind. Eng. Chem. Res., 42 (13): 2960–2967.

[41] Chen Z, Mathur VK (2003) Nonthermal Plasma Electrocatalytic Reduction of Nitrogen Oxide. Ind. Eng. Chem. Res. 42 (26): 6682–6687.

[42] Lin H, Huang Z, Shangguan W, Peng X (2007) Temperature-programmed oxidation of diesel particulate matter in a hybrid catalysis–plasma reactor. Proc. Combust. Inst 31: 3335–3342.

[43] Zhang PY, Liang FY, Yu G, Chen Q, Zhu WP (2003) A comparative study on decomposition of gaseous toluene by O_3_/UV, TiO_2_/UV and O_3_/TiO_2_/UV. J. Photochem. Photobiol. A: Chemistry 156: 189–194.

[44] Vandenbroucke AM, Morent R, Geyter ND, Leys C (2011) Decomposition of Trichloroethylene with Plasma-catalysis: A review. J. Hazard. Mater. 195: 30–54. 10.1016/j.jhazmat.2011.08.060 21924828

[45] Masuda S, Hosokawa S, Tu X, Sakakibara K, Kitoh S (1993) et.al (1993) Destruction of gaseous pollutants by surface-induced plasma chemical process (SPCS). IEEE Trans. Ind. Applicat. 29: 781–786.

[46] Yan K, Kanazawa S, Ohkubo T, Nomoto Y (1999) Oxidation and Reduction Processes During NO_x_ Removal with Corona-Induced Nonthermal Plasma. Plasma Chem. Plasma Proc. 19: 421–443.

